# Comparing typologies of violence exposure and associations with syndemic health outcomes among cisgender and transgender female sex workers living with HIV in the Dominican Republic

**DOI:** 10.1371/journal.pone.0291314

**Published:** 2023-09-11

**Authors:** Beth J. Maclin, Yan Wang, Carlos Rodriguez-Diaz, Yeycy Donastorg, Martha Perez, Hoisex Gomez, Clare Barrington, Deanna Kerrigan

**Affiliations:** 1 Department of Prevention and Community Health, Milken Institute School of Public Health, The George Washington University, Washington, District of Columbia, United States of America; 2 HIV Vaccine Trials Research Unit, Instituto Dermatológico y Cirugía de la Piel, Santo Domingo, Dominican Republic; 3 Department of Health Behavior, Gillings School of Global Public Health, University of North Carolina, Chapel Hill, North Carolina, United States of America; Universitat de Barcelona, SPAIN

## Abstract

Violence against women research largely excludes transgender women’s experiences and violence from perpetrators other than intimate partners. This study compares patterns of violence exposure among cisgender and transgender female sex workers (FSWs) and the associations with syndemic health outcomes. We used cross-sectional surveys from samples of cisgender and transgender FSWs living with HIV in the Dominican Republic (N = 211 and 100, respectively). We used latent class analysis to identify patterns of emotional, physical, and sexual violence and harassment by partners, clients, and police. We assessed sociodemographic and occupational predictors in relation to class membership, and class membership in relation to health (HIV continuum of care outcomes, mental health, substance use), using logistic regression. Two classes were identified in cisgender sample: Low Reported Violence Exposure (Class 1) and Sex Work-related Police Harassment (Class 2). Class 2 participants had greater odds of scoring abnormal or borderline abnormal anxiety on the Hospital Anxiety and Depression Scale (HADS-A) (adjusted OR = 3.97, p<0.01), moderate-to-severe depression per the Patient Health Questionnaire-9 (PHQ-9) (aOR = 5.74, p<0.01), and any illicit drug use in the past six months (aOR = 3.06, p<0.05), compared to Class 1. The transgender sample produced three classes: Low Reported Violence Exposure (Class 1); Sex Work-related Police Harassment (Class 2); and Sex Work-related Violence and Harassment (Class 3). Class 3 participants had greater odds of having anxiety (aOR = 6.65, p<0.01) and depression (aOR = 4.45, p<0.05), while Class 2 participants had greater odds of perfect ART adherence during the previous four days (aOR = 2.78, p<0.05), compared to Class 1. The more diverse and extreme violence patterns uncovered for the transgender sample show this group’s heightened risk, while similar patterns across groups regarding police abuse highlight a need for police-focused violence prevention interventions. Each sample’s highest violence class was associated with poor mental health, underscoring the need for mental health interventions for all FSWs.

## Introduction

Globally, sex workers experience a disproportionate burden of violence compared to the general population [1–6]. Social and structural factors, including sex work-related stigma and the criminalization of sex work, are significant drivers of violence against female sex workers (FSWs) across geographic settings [7–13]. Transgender FSWs have a further heightened vulnerability to, and greater likelihood of experiencing, violence compared to cisgender FSWs [4, 6, 14, 15]. Past research shows that gender identity is deeply intertwined with transgender FSWs’ vulnerability to abuse [6, 16]. Intersecting systems of oppression and marginalization, including sex work-related stigma and transphobia among others, make transgender FSWs particularly vulnerable to experiencing violence [4, 16–19].

In the Dominican Republic (DR), no law explicitly relates to a consenting adult aged 18 years or older engaging in sex work, making it de facto legal; there are laws, however, that restrict proxenetismo, which is when someone benefits financially from the exploitation, commercialization, or defrauding of another person in a sexual manner, and sex work by minors [20, 21]. The combination of sexism, transphobia, and limited legal systems to protect sex workers creates an environment where FSWs–both cisgender and transgender–face high rates of violence and harassment from state actors, intimate partners, and clients even though sex work is not illegal [22–26]. Among cisgender FSWs living with HIV in the DR, almost one in five (18%) reported having experienced intimate partner violence in the past six months [22]. Similarly, in a study by Hasbún [25], 21 percent of transgender FSWs reported experiencing physical violence by an intimate partner. Further, almost half of the respondents (42%) reported physical violence by clients, and a majority (71%) said they were discriminated against by the police [25].

Globally, FSWs are also at risk for and experience epidemics of HIV, substance use, and poor mental health that constitute a syndemic [27–29], resulting in part from the multiple forms of marginalization that FSWs endure. For FSWs living with HIV, various factors inform their ability to initiate and maintain HIV care and treatment, including substance use and violence exposure [22, 27]. Sex work-, HIV-, and violence victimization-related stigmas can negatively affect one’s mental health [30–32]. Further, violence victimization was found to have a positive bidirectional relationship with poor mental health and substance use [28, 30], and sexual violence victimization among FSWs is also associated with alcohol use [28].

Despite differences in risk and experiences of violence for cisgender and transgender FSWs [4, 6, 14, 15], few studies include and compare both groups; those that do largely focus on HIV and other sexually transmitted infections [14, 33, 34]. In addition, few studies have used latent class analysis (LCA) to assess the typologies of violence FSWs experience [35–37]; the three studies found that do use LCA either exclude transgender FSWs or do not provide any details regarding gender identity of participants. Typologies are a useful lens to examine violence exposure for FSWs because they can capture poly-victimization rather than looking at each incident, type, or perpetrator separately.

A gap exists in the current literature regarding typologies of violence experienced by transgender FSWs and how the transgender-specific typologies of violence exposure compare to those of cisgender FSWs. This study expands upon the violence against women (VAW) research cannon to identify and assess patterns of different forms of violence perpetrated by various actors experienced by cisgender and transgender FSWs living with HIV, specifically in the DR. To support the comparison between cisgender and transgender FSWs, we were guided by Intersectionality, a theory that acknowledges how people’s experiences, opportunities, and outcomes are informed by multiple systems of power and oppression that intersect in additive or compounding ways [38]. Furthermore, this study applies a syndemic framework to assess how those patterns of violence inform related and reinforcing health outcomes and identifies predictors of class membership.

We sought to answer the following questions: 1) what do patterns of violence from various perpetrators look like for cisgender and transgender FSWs?; 2) what factors are associated with risk of different patterns of violence exposure?; and 3) what is the relationship between different patterns of violence exposure and various health outcomes? We hypothesized that class structures would differ for cisgender and transgender FSWs; that sociodemographic and occupational factors would be associated with class membership; and that more severe patterns of violence exposure would be associated with poor HIV continuum of care, mental health, and substance use outcomes.

## Materials & methods

### Study design & sample

This study used two companion studies with cisgender and transgender FSWs living with HIV in Santo Domingo, DR. The parent study was prospective with three waves of data collection with cisgender FSWs in the DR as well as Tanzania [39]; the supplement study was cross-sectional and focused on transgender FSWs in the DR [40]. Both assessed how stigma and social cohesion influence HIV outcomes among FSWs living in HIV in the DR. Analyses were conducted on cross-sectional survey datasets of cisgender and transgender FSWs (N = 211 and 101, respectively). Neither of the samples were representative.

For the cisgender dataset, an existing cohort of FSWs from Abriendo Puertas, a multi-level intervention detailed elsewhere [41–43], was recruited by FSW peer navigators and through referrals from clinics, key informants, and participants themselves. We used the second wave of data, which was collected between December 2018 and November 2019, for this analysis.

For the transgender dataset, the same recruitment procedures were used led by FSW peer navigators re-engaging participants from an pilot adaptation study of the Abriendo Puertas intervention [44]. Data collection occurred between January and September 2019 [45].

All data collection was conducted in Santo Domingo, the capital of the DR, at the Instituto Dermatologico y Cirugia de la Piel (IDCP). All participants completed a survey of demographic, behavioral, and socio-structural factors. Further, a blood sample was collected for HIV viral load testing using polymerase chain reaction testing [41].

Both studies were reviewed and approved by the Johns Hopkins University (JHU) Bloomberg School of Public Health Institutional Review Board (IRB) and the IDCP. The George Washington University IRB deferred to the JHU IRB. Participants verbally consented to participate as approved by the JHU IRB and the IDCP. Participants were compensated for their time with $10 USD (paid in DR pesos).

### Measures

#### Violence items

Seven items capture participants’ experiences of violence [46–48]. These include: any physical and/or sexual violence from a steady partner; any physical and/or sexual violence from new clients; any physical and/or sexual violence from regular clients; forced to have sex with multiple clients in a group; any physical and/or sexual violence from police; any verbal harassment from police while exchanging sex for money; and any physical and/or sexual harassment from police while exchanging sex for money. Only participants who reported having a steady partner, new client, or regular client in the past 30 days were asked questions regarding physical and sexual violence in the past six months from each of these perpetrators. In order to keep all participants in the analysis, participants who answered “no” to the questions regarding partners and clients in the past 30 days were marked as “no/unknown” for the respective questions on physical and sexual violence in the past six months. Each item is a binary variable representing 1 = “yes” versus 0 = “no/unknown” or “no” to the violence exposure.

#### Predictor variables

*Socio-demographic variables*. These include: 1) age; 2) educational attainment based on the last grade completed by school level; 3) average monthly income from all revenue sources in the past six months; and 4) relationship status (currently partnered [married or living with partner] versus not [single/never married, divorced, separated, widowed]).

*Occupation variables*. These include: 1) traveling outside of the city where they reside to exchange sex for money in the past six months (yes versus no); 2) the average number of weekly clients over the past six months; 3) being drunk or high while exchanging sex for money (ever versus never); 4) where they most often meet clients (informal establishments only versus formal and informal establishments); and 5) where they most often go on dates with clients (informal establishments only versus formal and informal establishments). For the last two predictors, participants could select as many response options as necessary. Dichotomous variables were created for informal establishments only versus any formal establishments. For where participants meet clients, formal establishments include bars/clubs, hotel/guesthouse, and restaurants; informal establishments include private parties, on the street/park/outside, via mobile phone, corner stores, liquor stores, and car washes. For where participants go on dates with clients, formal establishments include bars/clubs and hotel/guesthouse; informal establishments include private parties, private vehicle, client’s house, participant’s house, other private house, and the street/park.

#### Syndemic health outcomes

*HIV continuum of care*. These include: 1) Viral suppression: defined as <400 copies/mL versus ≥400 copies/mL [49]; 2) Current ART use: yes versus no; 3) ART adherence: perfect adherence (i.e. no missed doses and all doses taken as instructed) during the previous four days versus non-perfect adherence; and 4) ART disruption: having stopped taking ART in the past six months: ever versus never.

*Mental health*. These include: 1) The anxiety-specific module from the Hospital Anxiety and Depression Scale (HADS-A), a 7-item scale, was utilized to assess symptoms of anxiety. The variable was categorized as Normal (score of 0–7) versus Borderline Abnormal or Abnormal (score of ≥8) [50]. 2) The Patient Health Questionnaire-9 (PHQ-9), a nine-item scale that assesses severity of depression on a 0–27 scale, was utilized to assess depressive symptoms. The variable categories include Moderate to Severe Depression (score of ≥10) versus Minimal to Mild Depression (score of 0–9) [51].

*Substance use*. These include: 1) The Alcohol Use Disorder (AUD) [52] measure is based on three questions about the frequency of drinking alcohol per week, the number of drinks consumed per event, and the frequency of consuming six or more drinks at a time; these comprise a 15-point scale. The variable categories include at risk for AUD (score of ≥3) versus not at risk (score of 0–2). 2) A measure was used to assess the frequency of illicit drug use, including marijuana, crack, cocaine, heroin, ecstasy, and other drugs. A variable was created to indicate use of any illicit drug versus none in the past six months.

### Analysis

Descriptive analyses were conducted for sociodemographic variables, occupational characteristics, and violence exposures. T-tests and chi-square statistical tests were conducted to compare the variables between the cisgender and transgender samples.

LCA was conducted within each group of participants to identify typologies of violence exposure. Additional classes were added to the model until fewer than five percent of participants were assigned to any given class. We used the following criteria to determine the best fitting number of classes: Akaike Information Criterion (AIC), Bayesian Information Criterion (BIC), and sample size-adjusted BIC (aBIC) (smaller values are better); Vuong-Lo-Mendell-Rubin Likelihood Ratio Test (VLMR-LRT) and Bootstrapped Likelihood Ratio Test (BLRT) (p-value<0.05, suggests that m classes fits the data better than m-1 classes); and entropy (greater than 0.70), as well as the meaningful conceptual interpretation of the latent classes [53–55]. We ran each model with 2,000 random starting values to ensure model identification. We compared the respective best fitting models for the cisgender and transgender samples and found different numbers of classes for each sample. The results of LCA are therefore presented for cisgender and transgender samples separately.

We assessed the relationship between predictors and class membership by conducting logistic regression or multinomial logistic regression, depending on the number of classes in the final models, using the three-step procedure in Mplus. To be parsimonious, the predictors with crude odds ratios with p-values<0.10 were included in the multivariate model.

Finally, we conducted logistic regression or multinomial logistic regression in Stata, depending on the number of classes, to examine the association between class membership and each syndemic health outcome, without and then with covariates that differed across classes. We conducted additional post-hoc regression analyses to examine potential syndemic-related explanations for individual health outcome results.

The descriptive analyses and outcome-related logistic regressions were conducted in Stata version 16.1 [56]. The LCA and predictor-related logistic regressions were conducted in Mplus (Version 8.7-Linux) [57]. Statistical significance was defined as p<0.05; marginal significance was defined as 0.05≤p<0.10.

## Results

### Group characteristics

Table 1 presents the summary of socio-demographics, occupation characteristics, syndemic health outcomes, and violence exposures of the study samples. The transgender participants earned more on average per month (mean 18,730 pesos vs. 9,640 pesos, p<0.001) and were more educated (i.e. 74.00% completed at least one grade level in secondary school or higher vs. 38.86%, p<0.001) than the cisgender participants. The cisgender participants were older (mean 40.91 years vs. 34.08 years, p<0.001) and a greater proportion were currently partnered (41.23% vs. 16.00%, p<0.001).

**Table 1:** Distribution and comparison of predictors, health outcomes, and violence exposures for cisgender and transgender samples (mean (SD) or frequency (%)).

	Cisgender (N = 211)	Transgender (N = 100)
Predictors		
Socio-Demographics		
Age (years)***	40.91 (8.93)	34.08 (9.96)
Relationship status***		
Currently partnered	87 (41.23%)	16 (16%)
Not partnered	124 (58.77%)	84 (84%)
Last school grade completed***		
None	9 (4.27%)	1 (1%)
Primary	120 (56.87%)	25 (25%)
Secondary	71 (33.65%)	52 (52%)
Post-secondary	11 (5.21%)	22 (22%)
Average monthly income from all income sources in past six months (DR pesos/1000)***	9.64 (6.64)	18.73 (17.01)
Occupation Characteristics		
Out of all the times you exchanged sex for money, how often were you drunk and/or high?***		
Ever	85 (40.28%)	74 (74%)
Never	126 (59.72%)	26 (26%)
In the past 6 months, have you traveled outside of the city where you reside specifically to exchange sex for money elsewhere?*		
Yes	32 (15.17%)	26 (26%)
No	176 (83.41%)	74 (74%)
Average number of clients per week during the past 6 months***	3.64 (2.98)	6.89 (5.38)
Place to meet clients		
Informal establishments only	170 (80.57%)	80 (80%)
Any formal establishments	41 (19.43%)	20 (20%)
Place to go on dates with clients		
Informal establishments only	40 (18.96%)	16 (16%)
Any formal establishments	171 (81.04%)	84 (84%)
Syndemic Health Outcomes		
Viral suppression (<400 copies/mL)*		
Yes	160 (75.83%)	64 (64%)
No	51 (24.17%)	36 (36%)
Currently taking ART***		
Yes	203 (96.21%)	84 (84%)
No/Missing	8 (3.79%)	16 (16%)
ART adherence in past 4 days		
Perfect adherence	84 (39.81%)	44 (44%)
Not perfect adherence	127 (60.19%)	56 (56%)
Stopped taking ART in past 6 months*		
Ever	54 (25.59%)	13 (13%)
Never/Missing	157 (74.41%)	87 (87%)
HADS-A		
Borderline Abnormal or Abnormal (≥8)	85 (40.28%)	34 (34%)
Normal (≤7)	126 (59.72%)	66 (66%)
PHQ-9		
Moderate to Severe Depression (≥10)	54 (25.59%)	24 (24%)
Minimal to Mild Depression (≤9)	157 (74.41%)	76 (76%)
Alcohol Use Disorder (AUD) Risk		
At risk	140 (66.35%)	77 (77%)
Not at risk	71 (33.65%)	23 (23%)
Illicit drug use in past 6 months***		
Any	27 (12.80%)	55 (55%)
None	184 (87.20%)	45 (45%)
Violence Exposures		
Any physical or sexual violence by a steady partner in the past 6 months		
Yes	10 (4.74%)	10 (10%)
No/Unknown	201 (95.3%)	90 (90%)
Any physical or sexual violence by a new client in the past 6 months***		
Yes	14 (6.64%)	20 (20%)
No/Unknown	197 (93.36%)	80 (80%)
Any physical or sexual violence by a regular client in the past 6 months**		
Yes	10 (4.74%)	15 (15%)
No/Unknown	201 (95.26%)	85 (85%)
In the past 6 months, have you been forced to have sex with multiple clients in a group?		
Yes	7 (3.32%)	7 (7%)
No	204 (96.68%)	93 (93%)
Any physical or sexual violence by the police in the past 6 months because of exchanging sex for money***		
Yes	3 (1.42%)	22 (22%)
No	208 (98.58%)	78 (78%)
Have you ever experienced verbal harassment from the police because of you exchanging sex for money?***		
Yes	31 (14.69%)	71 (71%)
No	180 (85.31%)	29 (29%)
Any physical or sexual harassment from the police because of exchanging sex for money***		
Yes	22 (10.43%)	57 (57%)
No	189 (89.57%)	43 (43%)

* = p<0.05,

**p<0.01,

*** = p<0.001.

P values are based on Chi-square tests for categorical variables or T-tests for continuous variable between the two groups.

For occupational characteristics, a larger proportion of transgender participants traveled for sex work (26.00% vs. 15.17%, p<0.05) and were ever drunk or high while exchanging sex for money (74.00% vs. 40.28%, p<0.001) than their cisgender counterparts. They also had more clients per week than the cisgender participants (mean score 6.89 vs. 3.64, p<0.001).

More cisgender FSWs were virally suppressed (75.83% vs. 64.00%, p<0.05) and currently taking ART (96.21% vs. 84.00%, p<0.001) compared to the transgender FSWs. Further, a larger proportion of cisgender participants reported ART disruption in the past six months compared to their transgender counterparts (25.59% vs. 13.00%, p<0.05). A larger proportion of transgender FSWs reported any illicit drug use in the past six months compared to the cisgender FSWs (55.00% vs. 12.80%, p<0.001).

For violence exposure, transgender FSWs reported more physical and/or sexual violence from new clients (20.00% vs. 6.64%, p<0.001), regular clients (15.00% vs. 4.74%, p<0.01), and police (22.00% vs. 1.42%, p<0.001) than the cisgender FSWs. Further, more transgender participants reported verbal harassment (71.00% vs. 14.69%, p<0.001) and physical and/or sexual harassment (57.00% vs. 10.43%, p<0.001) from police compared to the cisgender participants.

### Latent class analysis

#### Cisgender sample

Table 2 presents a summary of the models we assessed for the cisgender sample. Both the two-class and three-class models had satisfactory classification qualities, indicated by entropy >0.70. However, the two-class model was the best fit based on the BIC being smaller than the three-class model. Further the non-significant VLMR-LRT and BLRT comparing the three-class model to the two-class model indicates the three-class model did not provide significantly better fit than the two-class model. In addition, the smallest class proportion was less than five percent in the three-class model. The two-class model was therefore chosen as the final model.

**Table 2:** LCA model comparisons for cisgender and transgender samples (italics for selected model).

	2-Class Model	3-Class Model	4-Class Model
Cisgender Participants (N = 211)			
AIC	*579*.*655*	575.974	
BIC	*629*.*933*	653.067	
aBIC	*582*.*404*	580.188	
Vuong-Lo-Mendell-Rubin Likelihood Ratio Test for K-1 Classes	*<0*.*001*	0.3386	
Bootstrapped Likelihood Ratio Test for K-1 Classes	*<0*.*001*	0.0952	
Entropy	*0*.*89*	0.92	
Proportion for smallest class	*0*.*13697*	0.043	
Transgender Participants (N = 100)			
AIC	602.131	*591*.*917*	596.252
BIC	641.208	*651*.*836*	677.012
aBIC	593.835	*579*.*196*	579.106
Vuong-Lo-Mendell-Rubin Likelihood Ratio Test for K-1 Classes	0.0009	*0*.*0099*	0.1342
Parametric Bootstrapped Likelihood Ratio Test for K-1 Classes	0.00	*0*.*00*	0.3077
Entropy	0.809	*0*.*847*	0.881
Proportion for smallest class	0.33	*0*.*19462*	0.09

*Italics denote the class selected as best fitting the data.

The two classes are interpreted as Low Reported Violence Exposure (86.30% of participants) and Sex Work-related Police Harassment (13.70%). Table 3 presents the conditional probability of participants reporting each item for each class. For the Low Reported Violence Exposure class, none of the items exceed 0.05, suggesting very low likelihoods of participants reporting any violence or harassment in this class. For the Sex Work-related Police Harassment class, the probability of reporting any verbal or physical/sexual harassment from police is greater than 0.50 (0.82 and 0.60, respectively), while the probability of reporting the other violence items ranges between 0.10–0.38.

**Table 3:** Probability for each violence item and proportion of respondents by class for cisgender and transgender samples.

	Cisgender Sample (N = 211)		Transgender Sample (N = 100)		
	Sex Work- related Police Harassment	Low Reported Violence Exposure	Sex Work- related Violence and Harassment	Sex Work- related Police Harassment	Low Reported Violence Exposure
**Proportion of Sample**	13.70%	86.30%	19.46%	31.89%	48.65%
Any physical or sexual violence by a steady partner in the past 6 months	0.20	0.02	0.46	0	0.02
Any physical or sexual violence by a new client in the past 6 months	0.38	0.02	**0.70**	0.20	0
Any physical or sexual violence by a regular client in the past 6 months	0.30	0.01	**0.71**	0	0.02
In the past 6 months, have you been forced to have sex with multiple clients in a group?	0.24	0.00	0.29	0.05	0
Any physical or sexual violence by the police in the past 6 months because of exchanging sex for money	0.10	0.00	**0.70**	0.23	0.02
Have you ever experienced verbal harassment from the police because of you exchanging sex for money?	**0.82**	0.04	**0.89**	**1**	0.45
Any physical or sexual harassment from the police because of exchanging sex for money	**0.60**	0.03	**0.85**	**1**	0.18

**Items in bold** note when members of this class have greater than 0.5 probability of reporting this type of violence exposure.

Table 4 presents results for the predictors of class membership within the cisgender sample. In the crude models, significant positive predictors of Sex Work-related Police Harassment class membership, compared to the Low Reported Violence Exposure class, were: being drunk or high while exchanging sex for money (OR = 16.30; 95% CI 2.17, 122.47; p<0.01), average number of clients per week (OR = 1.16; 95% CI 1.04, 1.30; p<0.01), and traveling for sex work (OR = 2.69; 95% CI 0.90, 8.03; p<0.10). Meeting clients at only informal venues reduced the odds of cisgender FSWs being in the Sex Work-related Police Harassment class (OR = 0.27; (95% CI 0.10, 0.74; p<0.05). In the multivariate model, being drunk or high while exchanging sex for money increased the likelihood of being in the Sex Work-related Police Harassment class by roughly 10 times (adjusted OR = 10.11; 95% CI 1.93, 52.89; p<0.01). None of the other predictors were significant.

**Table 4:** Distribution of predictors across classes and tests of categorical latent variable logistic regressions using the 3-step procedure to identify predictors of class membership for cisgender sample with Low Reported Violence Exposure class as reference (mean (SD) or n (%)).

	**Sex Work-related Police Harassment**	**Low Reported Violence Exposure**	**Crude OR (95% CI)**	**p-value**
**Socio-Demographic Variables**				
Age (years)	38.73 (7.86)	41.21 (9.05)	0.96 (0.92, 1.01)	0.145
School level of last grade completed*			0.58 (0.14, 2.37)	0.450
None	3 (11.54%)	6 (3.24%)		
Primary	16 (61.54%)	104 (56.22%)		
Secondary	4 (15.38%)	67 (36.22%)		
Post-secondary	3 (11.54%)	8 (4.32%)		
Relationship status			1.62 (0.61, 4.35)	0.336
Currently partnered	13 (50%)	74 (40%)		
Not partnered	13 (50%)	111 (60%)		
Average monthly income from all income sources in past six months (DR pesos/1000)	9.51 (5.33)	9.65 (6.82)	1.00 (0.94, 1.05)	0.902
**Occupation Characteristic Variables**				
Out of all the times you exchanged sex for money, how often were you drunk and/or high?			16.30 (2.17, 122.47)	0.007***
Ever	21 (80.77%)	64 (34.59%)		
Never	5 (19.23%)	121 (65.41%)		
In the past 6 months, have you traveled outside of the city where you reside specifically to exchange sex for money elsewhere?			2.69 (0.90, 8.03)	0.076
Yes	7 (26.92%)	25 (13.51%)		
No	19 (73.08%)	160 (86.49%)		
Average number of clients per week during the past 6 months	5.23 (5.43)	3.42 (2.39)	1.16 (1.04, 1.30)	0.008***
Place to meet clients			0.27 (0.10, 0.74)	0.011**
Informal establishments only	16 (61.54%)	154 (83.24%)		
Any formal establishments	10 (38.46%)	31 (16.76%)		
Place to go on dates with clients			0.21 (0.02, 2.67)	0.231
Informal establishments only	2 (7.69%)	38 (20.54%)		
Any formal establishments	24 (92.31%)	147 (79.46%)		
**Multivariate Model**				
			**Adj. OR (95% CI)**	**p-value**
Out of all the times you exchanged sex for money, how often were you drunk and/or high?			10.11 (1.93, 52.89)	0.006**
Ever (vs. Never)				
Average number of clients per week during the past 6 months			1.08 (0.97, 1.21)	0.164
In the past 6 months, have you traveled outside of the city where you reside specifically to exchange sex for money elsewhere?			1.40 (0.31, 6.39)	0.664
Yes (vs. No)				
Place to meet clients			0.43 (0.11, 1.67)	0.222
Informal establishments only (vs. Any formal establishments)				

* = p<0.05,

** = p<0.01,

*** = p<0.001

Table 5 presents associations between class membership and syndemic health outcomes. Cisgender FSWs in the Sex Work-related Police Harassment class had roughly two times greater likelihood of ART disruption in the past six months than those in the Low Reported Violence Exposure class (aOR = 2.16; 95%CI 0.88, 5.31; p<0.10), adjusting for educational attainment. Further, those in the Sex Work-related Police Harassment class had almost four times greater likelihood of scoring Borderline Abnormal or Abnormal on the HADS-A (aOR = 3.97; 95%CI 1.60, 9.85; p<0.01), almost six times greater likelihood of having moderate to severe depression based on the PHQ-9 (aOR = 5.74; 95%CI 2.33, 14.11; p<0.001), and roughly three times greater odds of reporting illicit drug use in the past six months (aOR = 3.06; 95% CI 1.08, 8.71; p<0.05), compared to those in the Low Reported Violence Exposure class. Using a syndemic framework to better understand the relationship between ART disruption, violence exposure, and mental health, we conducted a post-hoc multiple logistic regression with anxiety and depression, as well as educational attainment, included as covariates. Sex Work-related Police Harassment class membership remained marginally associated with ART disruption compared to the Low Reported Violence Exposure class (aOR = 2.31; 95%CI 0.89, 5.97; p<0.10); none of the covariates were significant.

**Table 5:** Distribution of health outcomes across classes (n (%)) and association between class membership and health outcomes for cisgender sample with Low Reported Exposure class as reference.

	Low Reported Violence Exposure	Sex Work-related Police Harassment	Crude OR (95% CI)	p-value	Adjusted OR (95% CI)	p-value
**Proportion of participants**	**185 (87.68%)**	**26 (12.32%)**				
Viral suppression			0.85 (0.33, 2.15)	0.726		0.693
Yes	141 (76.22%)	19 (73.08%)			0.82	
No	44 (23.78%)	7 (26.92%)			(0.32, 2.15)	
ART adherence in past 4 days			0.78 (0.33, 1.83)	0.564		0.447
Perfect adherence	75 (40.54%)	9 (34.62%)			0.71	
Not perfect adherence	110 (59.46%)	17 (65.38%)			(0.29, 1.72)	
Stopped taking ART in past 6 months			2.00 (0.85, 4.73)	0.113		0.094
Ever	44 (23.78%)	10 (38.46%)			2.16	
Never	141 (76.22%)	16 (61.54%)			(0.88, 5.31)	
Currently taking ART			0.98 (0.11, 8.33)	0.988		0.991
Yes	178 (96.22%)	25 (96.15%)			0.99	
No	7 (3.78%)	1 (3.85%)			(0.11, 8.59)	
HADS-A			3.96 (1.64, 9.60)	0.002		0.003**
Borderline Abnormal | Abnormal	67 (36.22%)	18 (69.23%)			3.97	
Normal	118 (63.78%)	8 (30.77%)			(1.60, 9.85)	
PHQ-9			5.10 (2.17, 12.00)	0.000		0.000***
Moderate to severe depression	39 (21.08%)	15 (57.69%)			5.74	
Minimal to mild depression	146 (78.92%)	11 (42.31%)			(2.33, 14.11)	
Alcohol Use Disorder (AUD) Risk			1.81 (0.69, 4.72)	0.228		0.210
At risk	120 (64.86%)	20 (976.92%)			1.88	
Not at risk	65 (35.14%)	6 (23.08%)			(0.70, 5.05)	
Illicit drug use in past 6 months			3.04 (1.14, 8.12)	0.027		0.036*
Any	20 (10.81%)	7 (26.92%)			3.06	
None	165 (89.19%)	19 (73.08%)			(1.08, 8.71)	

* = p<0.05,

** = p<0.01,

*** = p<0.001

Covariate includes educational attainment as this differed significantly across classes.

#### Transgender sample

Table 2 presents a summary of the models tested for the transgender sample. The three-class model was the best fit based on the significant VLMR-LRT and BLRT for three classes and non-significant results for the four-class model. Further, the AIC and BIC are smaller for the three-class model compared to the four-class model, and the AIC and aBIC are smaller for the three-class model than the two-class model. The three classes are interpreted as Low Reported Violence Exposure (48.56% of participants), Sex Work-related Police Harassment (31.89%), and Sex Work-related Violence and Harassment (19.46%). Table 3 shows the conditional probabilities of participants reporting each item by class. For the Low Reported Violence Exposure class, all item probabilities are below 0.50. The probabilities of both of the two police harassment variables for the Sex Work-related Police Harassment class are 1.00. Finally, the variables on physical and/or sexual violence from new and regular clients and police, as well as both forms of harassment from police, are greater than 0.50, indicating this group is likely to experience violence and harassment from a variety of perpetrators.

Table 6 presents results for the predictors of class membership within the transgender sample. For each additional level of education completed, transgender FSWs have roughly 45% lower odds of being in the Sex Work-related Police Harassment class compared to the Low Reported Violence Exposure class (OR = 0.54; 95% CI 0.27, 1.08; p<0.10). Traveling for sex work increased the likelihood of transgender FSWs being in the Sex-work Related Violence and Harassment class (OR = 3.16; 95% CI 0.86, 11.64; p<0.10), while higher average monthly income reduced the likelihood of this class membership (OR = 0.95; 95% CI 0.91, 1.00; p<0.05), compared to the Low Reported Violence Exposure class. These three predictors remained statistically significant in the multivariate model. Educational attainment reduced the likelihood of being in the Sex Work-related Police Harassment class (aOR = 0.49; 95%CI 0.24, 0.98; p<0.05), while average monthly income reduced the likelihood of being in the Sex Work-related Violence and Harassment class (aOR = 0.93; 95%CI 0.88, 0.98; p<0.05). Sex work-related travel increased the likelihood of being in the Sex Work-related Violence and Harassment class (aOR = 5.32; 95%CI 1.18, 23.86; p<0.05).

**Table 6:** Distribution of predictors across classes and tests of categorical latent variable multinomial logistic regressions using the 3-step procedure to identify predictors of class membership for transgender sample with Low Reported Violence Exposure class as reference (mean (SD) or n (%)).

	**Sex Work-related Violence and Harassment**	**Sex Work-related Police Harassment**	**Low Reported Violence Exposure**	**Comparison Class**	**Crude OR (95% CI)**	**p-value**
**Socio-Demographic Variables**						
Age (years)	32.78 (9.01)	34.86 (10.70)	33.96 (9.84)	Sex work-related police harassment	1.01 (0.96, 1.06)	0.69
				Sex work-related violence and harassment	0.99 (0.93, 1.05)	0.645
School level of last grade completed				Sex work-related police harassment	0.54 (0.27, 1.08)	0.081
None	0 (0%)	1 (2.70%)	0 (0%)	Sex work-related violence and harassment	0.87 (0.37, 2.01)	0.735
Primary	4 (22.22%)	12 (32.43%)	9 (20%)			
Secondary	10 (55.56%)	18 (48.65%)	24 (53.33%)			
Post-secondary	4 (22.22%)	6 (16.22%)	12 (26.67%)			
Relationship status				Sex work-related violence and harassment	2.56 (0.49, 13.53)	0.268
Currently partnered	4 (22.22%)	7 (18.92%)	5 (11.11%)	Sex work-related police harassment	2.04 (0.47, 8.82)	0.342
Not partnered	4 (22.22%)	7 (18.92%)	5 (11.11%)			
Average monthly income from all income sources in past six months (DR pesos/1000)***	12.25 (6.44)	21.36 (22.71)	19.15 (13.77)	Sex work-related police harassment	1.01 (0.98, 1.03)	0.613
				Sex work-related violence and harassment	0.95 (0.91, 1.00)	0.027*
**Occupation Characteristic Variables**						
Out of all the times you exchanged sex for money, how often were you drunk and/or high?				Sex work-related police harassment	1.53 (0.51, 4.61)	0.451
				Sex work-related violence and harassment	1.05 (0.27, 4.06)	0.938
Ever	13 (72.22%)	29 (78.38%)	32 (71.11%)			
Never	5 (27.78%)	8 (21.62%)	13 (28.89%)			
Average number of clients per week during the past 6 months**	6.67 (3.74)	7.54 (7.09)	6.44 (4.24)	Sex work-related police harassment	1.04 (0.96, 1.13)	0.355
				Sex work-related violence and harassment	1.01 (0.92, 1.12)	0.823
In the past 6 months, have you traveled outside of the city where you reside specifically to exchange sex for money elsewhere?				Sex work-related police harassment	0.96 (0.30, 3.08)	0.946
Yes	8 (44.44%)	8 (21.62%)	10 (22.22%)	Sex work-related violence and harassment	3.16 (0.86, 11.64)	0.084
No	10 (55.56%)	29 (78.38%)	35 (77.78%)			
Place to meet clients				Sex work-related police harassment	1.75 (0.53, 5.79)	0.358
				Sex work-related violence and harassment	1.69 (0.35, 8.26)	0.515
Informal establishments only	15 (83.33%)	31 (73.78%)	34 (75.56%)			
Any formal establishments	3 (16.67%)	6 (15.22%)	11 (24.44%)			
Place to go on dates with clients				Sex work-related police harassment	1.09 (0.31, 3.79)	0.893
				Sex work-related violence and harassment	0.19 (0.01, 5.48)	0.329
Informal establishments only	1 (5.56%)	7 (18.92%)	8 (17.78%)			
Any formal establishments	17 (94.44%)	30 (81.08%)	37 (82.22%)			
**Multivariate Model**						
				**Comparison Class**	**Adj. OR (95% CI)**	**p-value**
School level of last grade completed				Sex work-related police harassment	0.49 (0.24, 0.98)	0.045*
				Sex work-related violence and harassment	1.28 (0.43, 3.81)	0.664
Average monthly income from all income sources in past six months (DR pesos/1000)				Sex work-related police harassment	1.01 (0.99, 1.04)	0.267
				Sex work-related violence and harassment	0.93 (0.88, 0.98)	0.011*
In the past 6 months, have you traveled outside of the city where you reside specifically to exchange sex for money elsewhere?				Sex work-related police harassment	0.91 (0.26, 3.19)	0.885
Yes (vs. No)				Sex work-related violence and harassment	5.32 (1.18, 23.86)	0.029*

* = p<0.05,

** = p<0.01,

*** = p<0.001

Table 7 presents the associations between class membership and health outcomes for the transgender sample, adjusting for average monthly income. Those in the Sex Work-related Police Harassment class had almost three times greater likelihood of perfect ART adherence (aOR = 2.78; 95%CI 1.12, 6.88; p<0.05) and almost 2.5 times greater odds of having borderline abnormal or abnormal anxiety (aOR = 2.40; 95% CI 0.89, 6.45; p<0.10), compared to those in the Low Reported Exposure class. Transgender FSWs in the Sex Work-related Violence and Harassment class had almost seven times greater likelihood of scoring Borderline Abnormal or Abnormal on the HADS-A (aOR = 6.65; 95%CI 1.98, 22.37; p<0.01) and nearly five times greater likelihood of having moderate to severe depression based on the PHQ-9 (aOR = 4.45; 95%CI 1.28, 15.47; p<0.01), compared to those in the Low Reported Violence Exposure class.

**Table 7:** Distribution of health outcomes across classes (n (%)) and association between class membership and health outcomes for transgender sample with Low Reported Exposure as reference.

	Low Reported Violence Exposure	SW-related Police Harassment	SW-related Violence & Harassment	Comparison Class	Crude OR (95% CI)	p-value	Adjusted OR (95% CI)	p-value
**Proportion of participants**	**45 (45%)**	**37 (37%)**	**18 (18%)**					
Viral suppression				SW-related Police Harassment	0.81 (0.33,1.98)	0.643	0.79 (0.32,1.95)	0.612
				SW-related Violence & Harassment	1.43 (0.43,4.76)	0.555	1.56 (0.46,5.23)	0.475
Yes	29 (64.44%)	22 (59.46%)	13 (72.22%)					
No	16 (35.56%)	15 (40.54%)	5 (27.78%)					
ART Adherence in past 4 days				SW-related Police Harassment	2.66 (1.08,6.51)	0.033	2.78 (1.12,6.88)	0.028*
				SW-related Violence & Harassment	0.91 (0.29,2.88)	0.867	0.82 (0.26,2.64)	0.744
Perfect adherence	16 (35.56%)	22 (59.46%)	6 (33.33%)					
Not perfect adherence	29 (64.44%)	15 (40.54%)	12 (66.67%)					
Stopped taking ART in past 6 months				SW-related Police Harassment	0.85 (0.26,2.93)	0.795	0.72 (0.19,2.65)	0.616
				SW-related Violence & Harassment	0.32 (0.04,2.80)	0.303	0.41 (0.05,3.66)	0.423
Ever	7 (15.56%)	5 (13.51%)	1 (5.56%)					
Never	38 (84.44%)	32 (86.49%)	17 (94.44%)					
Currently taking ART				SW-related Police Harassment	2.06 (0.58,7.74)	0.264	2.05 (0.58,7.31)	0.267
				SW-related Violence & Harassment	1.25 (0.30,5.27)	0.761	1.27 (0.30,5.48)	0.745
Yes	36 (80%)	33 (89.19%)	15 (83.33%)					
No	9 (20%)	4 (10.81%)	3 (16.67%)					
HADS-A				SW-related Violence & Harassment	6.29 (1.90,20.80)	0.003	6.65 (1.98,22.37)	0.002**
Borderline Abnormal or Abnormal	9 (20%)	14 (37.84%)	11 (61.11%)	SW-related Police Harassment	2.43 (0.91,6.54)	0.077	2.40 (0.89,6.45)	0.084
Normal	36 (80%)	23 (62.16%)	7 (38.89%)					
PHQ-9				SW-related Police Harassment	1.50 (0.49,4.61)	0.481	1.48 (0.47,4.67)	0.508
Moderate to severe depression	7 (15.56%)	8 (21.62%)	9 (50%)	SW-related Violence & Harassment	5.43 (1.59,18.50)	0.007	4.45 (1.28,15.47)	0.019*
Minimal to mild depression	38 (84.44%)	29 (78.38%)	9 (50%)					
Alcohol Use Disorder (AUD) Risk				SW-related Police Harassment	1.67 (0.55,5.06)	0.363	1.70 (0.55,5.22)	0.358
				SW-related Violence & Harassment	0.65 (0.20,2.13)	0.474	0.77 (0.23,2.59)	0.668
At risk	34 (75.56%)	31 (83.78%)	12 (66.67%)					
Not at risk	11 (24.44%)	6 (16.22%)	6 (33.33%)					
Illicit drug use in past 6 months				SW-related Police Harassment	1.03 (0.43,2.46)	0.948	1.01 (0.42,2.43)	0.976
				SW-related Violence & Harassment	1.38 (0.45,4.19)	0.575	1.45 (0.47,4.48)	0.517
Any	24 (53.33%)	20 (54.05%)	11 (61.11%)					
None	21 (46.67%)	17 (45.95%)	7 (38.89%)					

* = p<0.05,

** = p<0.01,

*** = p<0.001

Covariate included average monthly income as this differed significantly across classes..

## Discussion

We defined and compared violence exposure patterns for cisgender and transgender FSWs and uncovered unique relationships between these group-specific violence exposure typologies and other syndemic health outcomes. While we found similarities between the two groups, suggesting that the shared intersecting systems of oppression they face—sexism, sex work-related stigma, and HIV-related stigma—inform the types and severity of violence exposure, concrete differences highlight how transphobia informs greater risk for transgender FSWs in the DR.

The frequencies for each violence item show that transgender FSWs experience more violence than their cisgender counterparts, which mirrors past research [58–60]. While these individual variables are useful in beginning to understand violence exposure among FSWs, they do not allow for examination of how these forms of violence co-exist. When looking at these items together through LCA, we elucidate different patterns of violence exposure for the cisgender and transgender samples. Not only did the best fitting model for the transgender sample have an additional class—Sex Work-Related Violence and Harassment—but that class includes more severe forms of violence by multiple perpetrators. In addition, the two conceptually comparable classes across samples—Low Reported Violence Exposure and Sex Work-related Police Harassment—are not equivalent. The transgender FSWs assigned to the latter class have an estimated 100 percent probability of experiencing both verbal harassment and physical and/or sexual harassment from police, compared to 81.5 percent and 60 percent, respectively, for the cisgender sample. For the Low Reported Violence Exposure classes, none of the items go above five percent for the cisgender sample, but verbal harassment from police approaches 50 percent, suggesting that even those assigned to the lowest violence exposure class have different risks of exposure based on transphobia. Further, while less than 15 percent of the cisgender participants were assigned to the highest violence class in their respective model (Sex Work-related Police Harassment), more than half of the transgender participants are in a class that suggests a high probability of experiencing at least verbal, physical, and/or sexual harassment from police while exchanging sex for money.

These models support existing evidence that transgender women in the DR face systemic gender-based violence (GBV) partly due to transphobia [24, 25, 61–63]. In a study of transgender FSWs in the DR, most respondents said they felt they were discriminated against more because they were transgender than because they were sex workers [25]. The lack of legal protection against discrimination or violence based on gender identity, widely accepted transphobic beliefs, and sexist gender norms, create a storm of state indifference, police abuse, and extensive discrimination in employment, health care, and housing [23, 24, 26, 64, 65]. These collectively translate into an environment that fosters violence.

Another important finding from both the cisgender and transgender models is the prominent role of police in creating an unsafe environment for FSWs. While sex work in the DR is not illegal for consenting adults, which all participants were, reports show that police routinely harass, rob, and abuse sex workers with no legal cause [23, 66]. These models show how types of police harassment and violence co-occur and that, regardless of gender identity, a sizable group of FSWs have a high probability of experiencing sex work-related police harassment. Further, past research shows how police violence cascades to create an environment conducive to violence against FSWs from various perpetrators. First, police abuse their position of power by threatening or enacting arrest, detainment, and violence to coerce FSWs into sexual acts often referred to as or considered “free services” [13, 16, 67–69]. Victims of police violence are unable or unlikely to report these crimes to the state institution where the perpetrators work for fear of further abuse or being discriminated against, creating a climate of impunity for police [16, 67]. As a result of police perpetrating violence, FSWs may also not feel safe or supported to report crimes perpetrated by others, such as clients, which fosters an environment of impunity for all perpetrators [2, 67, 68, 70].

One possible explanation for the configuration of Sex Work-related Violence and Harassment class from the transgender model, which shows high probability of experiencing physical and/or sexual violence from new and regular clients in addition to all forms of harassment and violence from police, is how police behavior can impact FSWs’ work decisions and the implications for violence exposure. Shannon et al. [71] reported that both prior assault by police and changing working areas to less populated streets because of more active policing on main streets increased the odds of all FSWs experiencing client perpetrated violence. A systematic review found that police financially extort sex workers under the threat of arrest and physical and sexual violence, which leads sex workers to take on greater risk from clients and forms of sex to compensate for the financial loss [68]. Specific to transgender FSWs, Ganju & Saggurti [16] reported that police harassment reduced their choices with regards to clients, condom use, and work space. Similar results were found by Mukherjee et al., [35], who reported that police violence victimization was associated with having more clients, economic incentives for condomless sex, and client violence victimization among FSWs who use drugs; while not specific to transgender FSWs, these associations are still relevant given the high proportion of transgender FSWs in this study who reported illicit drug use in the past six months and were at risk for AUD. When considering the occupational characteristics of the two samples, we found a difference in the number of weekly clients transgender and cisgender FSWs reported, with the former reporting significantly more than the latter. This could suggest that transgender FSWs are both working more, perhaps trying to make up for financial losses resulting from police abuse, and taking on riskier clients. More research is needed to better understand the ecology of sex work in which cisgender and transgender FSWs operate.

This study has important implications for violence prevention among FSW. Violence prevention interventions are needed in the DR to change police engagement with FSWs. Work in India under the umbrella of the Avahan program, which was launched as a 10-year initiative to change the course of the HIV epidemic in India [72], offers examples of how this can be done and the impact of such efforts. Ashodaya Samithi is a sex workers’ organization in Mysore, India, based on “community-led structural intervention elements that focus on community mobilization, increased access and utilization of services, and creating an enabling environment” [73, p. 70]. Their activities addressed multiple sources of violence, including boyfriends, lodge owners, community leaders, and police [73, 74]. Through their multi-pronged community-led structural intervention, which involved the creation of a safe space for FSWs, Crisis Intervention Teams, and advocacy efforts, police violence against FSWs decreased substantially [74]; further, police support and protection increased [73]. Similar multi-pronged efforts to both hold police accountable for their behavior as state actors through policy and monitoring of laws as well as to change their beliefs regarding sex work and gender identity should be considered in the DR context.

Beyond the direct impact of violence, we must consider the broader role of harassment and violence on syndemic health outcomes. We found that being assigned to the respective class with the greatest violence exposure pattern increased the odds of both cisgender and transgender FSWs scoring Borderline Abnormal or Abnormal on the HADS-A and having moderate to severe depression based on the PHQ-9. Exposure to violence from intimate or regular partners, clients, police, and others is significantly and directly associated with poor mental health outcomes for FSWs around the world [30, 75–79]. Other work using LCA to define patterns of GBV among cisgender FSWs in Kenya found comparable results that participants assigned to the most severe violence class had significantly greater odds of having moderate to severe depression as well as post-traumatic stress disorder [37].

Additionally, being in the Sex Work-related Police Harassment class was significantly associated with HIV continuum of care for both groups, though not entirely as expected. For the cisgender sample, being in the Sex-work Related Police Harassment class was marginally associated with greater odds of ART disruption in the past six months. This finding mirrors past research that shows police harassment, and GBV more broadly, can impede successful HIV treatment among sex workers [68, 80–82]. One potential explanation is the negative impact of poor mental health on HIV care engagement and maintenance [83–86]. However, anxiety and depression were both non-significant covariates in the post-hoc multiple logistic regression we conducted examining this relationship. Future research is needed to understand the specific mechanisms through which police harassment informs ART disruption among cisgender FSWs in the DR.

In contrast, the association between violence and HIV care for the transgender sample was the opposite of what was expected based on the existing research and compared to the cisgender sample. Transgender participants assigned to their respective Sex Work-related Police Harassment class had significantly greater odds of perfect ART adherence during the previous four days. It is possible that the fact that the transgender women were recruited for this study in part through HIV clinics and by peer navigators from a pilot intervention focused overall on improving HIV care and treatment helps explain this result [81]. However, additional work, particularly qualitative research, is needed to understand the dynamics between police harassment and positive HIV treatment outcomes for transgender FSWs. There may also be differences regarding the medication regimen prescribed to transgender versus cisgender FSWs that may inform this difference; future work should include more detailed questions regarding exact ART prescriptions to elucidate the dynamics at play that may explain this unexpected result regarding adherence.

In addition to considering how violence exposure patterns relate to syndemic health outcomes, we identified significant predictors of class membership. The only consistent predictor variable for both groups was traveling to exchange sex for money, which increased the odds of being in each group’s respective highest violence exposure class. Sex work-related mobility is often framed as a risk factor, as it has been found to increase FSWs’ odds of experiencing violence as well as increasing the severity of violence [87, 88]. Mobility, however, is not entirely negative as it is also a manifestation of a FSW’s agency and autonomy and can represent new social and economic opportunities [87, 89]. Qualitative methods would be particularly useful to uncover the dynamics of how sex work-related migration increases the odds of sex work-related police harassment for cisgender FSWs and sex work-related violence and harassment for transgender FSWs.

A consistent divergence found between the cisgender and transgender samples, for both predictors of and outcomes associated with class membership, relates to substance use. For the predictors, ever being high or drunk while exchanging sex for money was significantly associated with being in the respective highest violence exposure class, but only for the cisgender FSWs. For the outcomes, being in the sample-specific highest violence exposure class was associated with greater odds of illicit drug use in the past six months, but, again, only for the cisgender FSWs. These results from the cisgender sample mirror existing research that shows that substance use while working increases the odds of experiencing violence from clients and police [90–92] and violence victimization increases the odds of substance use among FSWs [28, 30, 93, 94]. Rather than understanding these results as suggesting FSWs who experience violence are at fault, they offer important insights into the context in which violence is perpetrated against FSWs and help identity potential intervention points for harm reduction. In Tanzania, cisgender FSWs who work in bars worked together to address or subvert threats of violence associated with alcohol consumption throughout the sex exchange process [95]. This included coordinating with barmaids to be given water in beer bottles when potential clients buy them drinks in order to remain sober. During negotiations with clients, participants described how they would pool money if another sex worker was unable to pay back a refused client for the drinks he purchased as a way to mitigate the risk of violence [95].

For the transgender sample, the lack of significant associations with both variables related to substance use is unexpected based on this existing literature connecting sex work, substance use, and violence victimization [28, 30, 90–94]. We may have found null results because both sex work-related substance use and illicit drug use among the transgender sample was high regardless of class membership and significantly different from the cisgender sample, suggesting substance use behavior is prevalent among transgender FSWs living with HIV in the DR. The lack of variance across classes might instead suggest that high substance use, particularly illicit drug use, among this group may be more informed by the additional layer of oppression in the form of transphobia and its intersections with sex work-related stigma, HIV-related stigma, and sexism. Additional research is needed to understand the relationships between substance use, violence, and different modes of oppression for transgender FSWs.

Regardless of the differences found between the samples, the combination of high reported substance use among transgender FSWs and significant differences in substance use based on violence exposure patterns for cisgender FSWs highlight the need for interventions related to substance use among all FSWs. Existing research shows that FSWs’ inability to access substance use treatment is significantly associated with different forms of GBV, including violence and harassment from clients and police [96, 97]. Pursuing interventions that address substance use treatment access may then also mitigate violence exposure, though research and evaluation of these connections are needed.

### Limitations

Despite the strengths of using LCA, comparing cisgender and transgender FSWs, and including multiple violence and harassment items, this study has limitations. One is the use of cross-sectional surveys. Another is the relatively small non-representative sample sizes, which means the results are not generalizable. The sample sizes were calculated for the primary research questions, not for conducting a LCA. Concerns about power may exist, particularly when examining the transgender sample, which only has 100 participants. However, we limited the number of items included in the model as smaller sample sizes may be adequate for models with fewer items and classes [98]. Further, Muthén [99] notes that rules of thumb for minimum sample sizes are not as important for mixture models, such as LCA, and that greater attention should be paid to the specifics of the model and sample at hand and how well the model fits the data. Thus, given the strength of the criteria used to identify the best fitting models, concerns about sample size are noted, but should not negate the results presented.

Further, this secondary analysis draws from two studies that were not specifically designed to examine experiences of violence among FSWs. There may be other forms of violence that FSWs experience or nuances regarding those experiences (i.e. where it occurs, when it occurs, who else is around, etc.) that are missing. Additionally, a skip pattern in the survey led to only participants that reported having specific partner types in the previous 30 days being asked about physical and sexual violence related to those types of partners in the past six months. This skip logic, combined with the fact that intimate partner violence and sexual violence are often underreported [100, 101], means that the frequencies for regular partner and new and regular client violence likely underestimate the true exposure. Thus, the patterns of violence exposure may miss or underrepresent regular partner and client physical and sexual violence. While the patterns reported here reflect the data collected and are well supported, it should not be assumed that participants from either sample do not experience violence from clients and regular partners.

Additionally, differences between the cisgender and transgender FSWs are ascribed to transphobia, yet we do not include any measures of transphobia in the analyses conducted. Rather, we assume that systemic oppression based on transgender identity is a key driver of the differences found between the two groups of women given the overlap they share on the other forms of systemic oppression that could otherwise explain differences in violence exposure, i.e. sex work-related stigma, HIV-related stigma, and sexism. Intersectionality helps us understand the differential impact of transphobia on the experiences of oppression and marginalization for transgender and cisgender FSWs. We, however, did not conduct an intersectional analysis; this would be a valuable pursuit for future research. It is also possible there are other aspects of systemic oppression at play that are not considered or measured in this analysis. Finally, we utilize a syndemic framework to consider various health outcomes and how they may interact with each other, but we do not conduct a syndemic analysis. Instead of testing disease interaction [102, 103], we added specific health outcomes to post-hoc analyses when deemed appropriate to examine how some health outcomes may influence others.

## Conclusion

This study presents a nuanced understanding of the nature of violence exposure for FSWs living with HIV in the DR and illuminates how transphobia informs violence severity for cisgender and transgender FSWs differently. Further, police were identified as key perpetrators for both groups, suggesting that interventions targeting police interactions with FSWs are needed as these state actors significantly impact different syndemic health outcomes for both cisgender and transgender FSWs. Finally, more extreme patterns of violence for each group were significantly associated with anxiety and depression, which signals a need for greater investment in mental health care for the entire FSW community.
